# Dental Antibiotic Prescribing Practices and Antimicrobial Resistance Knowledge in Singapore

**DOI:** 10.1016/j.identj.2026.109654

**Published:** 2026-06-03

**Authors:** Jia Qi Low, Marietta Taylor, Behnjemyn Jeng Kit Loh, Jacob Ren Jie Chew, Leanne Teoh, Charlene Enhui Goh

**Affiliations:** a Faculty of Dentistry, National University of Singapore, Singapore; b Melbourne Dental School, University of Melbourne, Victoria, Australia

**Keywords:** Antimicrobial stewardship, Drug resistance, Inappropriate prescribing, Knowledge and attitudes, Antibiotics, Antimicrobial resistance

## Abstract

**Introduction and aims:**

While dental antibiotic prescribing accounts for approximately 10% of all antibiotic prescriptions across human healthcare worldwide, up to 80% are inappropriate. These contribute to the growing global health threat of antimicrobial resistance (AMR). Effective antibiotic stewardship requires an up-to-date understanding of antibiotic prescribing practices and knowledge. This study aimed to assess the antibiotic prescribing practices, knowledge and attitudes among dentists in Singapore.

**Methods:**

A survey was distributed in 2024 to dentists using convenience sampling through the mailing lists of professional dental organisations. Appropriate use was assessed by comparing the responses with dental prescribing guidelines and stratified by clinical specialty. Multivariable logistic regressions were used to identify dentist characteristics predicting appropriate prescribing.

**Results:**

Two hundred and eighty dentists completed the questionnaire. Appropriate antibiotic prescribing varied widely, ranging from 6.5% to 97.7% across clinical scenarios. The lowest appropriate prescribing rates were observed for periodontal scenarios (30.4% ± 37.5%). Rates were similarly low for oral surgery scenarios (34.0% ± 29.0%) but higher for restorative (58.3% ± 28.6%) and for the management of medically complex patients (56.6% ± 18.0%). When faced with nonclinical pressures (eg, patient request for antibiotics, lack of a definitive diagnosis), only 21.1% to 37.5% practised appropriate prescribing. In multivariable regressions, locally trained dentists, those in the public sector, and those with fewer years of experience showed higher rates of appropriate prescribing.

**Conclusions:**

Although dentists recognised the importance of appropriate antibiotic use and the threat of AMR, many demonstrated inappropriate prescribing and gaps in their understanding of AMR. Future training interventions could increase awareness of recently published local guidelines and offer digital resources to address knowledge gaps and meet training demand. Implementation strategies are also needed to translate interventions into practice.

**Clinical Relevance:**

Understanding antibiotic prescribing practices, knowledge, and attitudes among dentists in Singapore can guide targeted interventions to enhance antibiotic stewardship within the dental profession.

## Introduction

Antimicrobial resistance (AMR) is a critical global public health threat. In 2019, 1.27 million deaths worldwide were caused by antimicrobial-resistant bacterial infections, with an additional 4.95 million deaths associated with AMR-related complications.^1^ As antimicrobial-resistant infections become increasingly challenging to treat, patients experience prolonged hospital stays and higher morbidity and mortality rates, placing greater strain on healthcare systems.^2^ By 2050, AMR is estimated to cost the global economy as much as $3 trillion in lost gross domestic product.^2^

Inappropriate and excessive use of antibiotics in healthcare, is one of the key drivers of this global health crisis.^3^ In an Australian survey, approximately 55% of antibiotics were reported to be inappropriately prescribed by dentists, such as in the management of localised odontogenic infections.^4^ With the dental profession responsible for around 10% of antibiotic prescriptions across human healthcare worldwide,^5^ dentists play a key role in appropriate prescribing and thus combating AMR. Yet, several international surveys have reported significant gaps in the profession’s literacy on antibiotic use, and concerning levels of inappropriate prescribing of up to 80%.^6^, ^7^, ^8^, ^9^ These findings highlight the need for stronger antibiotic stewardship interventions in dentistry worldwide.

A recent 2024 systematic review of studies across multiple countries found that inappropriate prescribing in dentistry is mainly driven by modifiable factors such as inadequate knowledge, complacency towards patient expectations, time pressure, and fear of adverse patient outcomes and medico-legal complications.^9^ However, the review identified no studies from Singapore, revealing a lack of local data. The last Singapore-based study examining antibiotic prescription practices was conducted a decade ago, and focused only on antibiotic prophylaxis for the prevention of infective endocarditis.^10^ The larger patterns of antibiotic use in routine dental practice are unknown. Addressing these knowledge gaps is essential to inform and design targeted educational interventions to support effective antibiotic stewardship in dentistry in Singapore.

This study aims to assess the current antibiotic prescribing practices and knowledge of antibiotic use among dentists in Singapore, and to examine how dentist characteristics influence prescribing behaviour. We further explore the factors that shape prescribing decisions, assess dentists’ knowledge and attitudes toward antimicrobial resistance, and identify their sources of knowledge and interest in additional training on antibiotic use. The findings of this study will inform the design of effective future interventions to strengthen antibiotic stewardship among dentists.

## Materials and methods

### Study design and population

This cross-sectional study was conducted in 2024 among registered dentists in Singapore using convenience sampling. A self-administered online survey questionnaire (Qualtrics©) was distributed through the mailing lists of professional dental organisations, including the Singapore Dental Association (SDA) and College of General Dental Practitioners Singapore (CGDP). These are the two largest professional dental societies in Singapore. Additional dissemination was carried out through each of the dental specialist associations in Singapore. The study team did not access or obtain any individual contact information from the professional organisations. Participation was voluntary, informed consent was obtained electronically from all participants, and all responses were anonymous.

### Sample size calculation

As the prevalence of appropriate prescribing among dentists in Singapore has not been previously reported, a conservative estimate of 50% was assumed for sample size calculation. Based on the population of 2690 registered dentists in Singapore,^11^ a minimum sample size of 337 respondents was needed to achieve a 95% confidence level with a 5% margin of error.

### Questionnaire

The questionnaire was based on previous surveys in the literature^12^, ^13^, ^14^, ^15^ and modified for the local context. The questionnaire comprised of 5 sections: (1) Demographics (6 items), (2) Antibiotic prescribing preferences (4 items), (3) Prescribing practices for clinical indications and prophylaxis; and nonclinical scenarios (29 items), (4) Knowledge and attitudes towards antimicrobial resistance (7 items), and (5) Sources of prescribing knowledge and training needs (4 items). The detailed survey questions are available in Supplementary Table 1.

Demographic information, including age , sex, location of basic dental training (Singapore or overseas-trained), main practice sector (private or public sector), years of clinical experience, and clinical specialty (Q1-Q6) was obtained.

Characteristics of antibiotic prescribing preferences were explored by identifying the antibiotic most often prescribed, the most common prescription duration, the frequency of prescribing a multidrug regimen, and the combination of antibiotics prescribed in a multidrug regimen (Q7-Q10).

Dentists were asked about their prescribing preferences for clinical and nonclinical scenarios. Some examples of questions include: ‘Please indicate how often you prescribe a course of antibiotics for treating peri-implant infections, in addition to a dental treatment’, and ‘For which of the following patients would you prescribe preoperative prophylactic antibiotics before starting invasive dental treatment – mitral valve prolapse with valvular regurgitation’. Clinical scenarios were categorised according to their clinical context: restorative, oral surgery, periodontal and implant-related, and the management of medically complex patients. The appropriateness of prescription was based off an earlier study,^4^ local guidelines^16^ and individual references relevant to the specific clinical scenario^17^, ^18^, ^19^, ^20^, ^21^, ^22^, ^23^, ^24^ (Supplementary Table 3). Each dentist’s appropriate prescribing rate in these clinical scenarios was scored and tabulated for each category. The nonclinical scenarios explored common pressures experienced by dentists, consisting of patient expectations, time pressure, ineffective local anaesthetic, inability to arrive at a diagnosis and medico-legal conflicts (Q15). Dentists were also surveyed on their knowledge and attitudes towards AMR. For example, participants were asked to indicate whether ‘Prescribing antibiotics for longer than the recommended duration does not contribute to antibiotic resistance’ and ‘Antibiotic-resistant bacteria spread easily from person to person’ were true or false.

Finally, to inform the development of interventions to improve antibiotic practices, we examined sources of prescribing knowledge and training needs. Dentists were asked about their prior antibiotic training, their primary sources of prescribing information, their willingness to receive further training, and their views on the effectiveness of various potential measures to promote appropriate prescribing.

### Statistical analysis

Descriptive statistics on prescribing preferences, patterns and appropriate use are presented as frequencies, means, and standard deviation (SD). For each respondent, the percentage of appropriate prescribing in each clinical category of clinical indication (restorative, oral surgery, periodontal, and medical complex patients) was calculated, excluding ‘Do not do procedure’ responses. Independent samples *T*-tests or one-way analysis of variance (ANOVA) with Bonferroni adjustments were used to compare appropriate prescribing rates across clinician characteristics (years of clinical experience, sex, location of basic dental training and main practice sector).

To gain further understanding of the dentist characteristics predicting appropriate prescribing for specific clinical scenarios, associations between dentist characteristics and appropriate prescribing for each clinical scenario were evaluated using multivariable logistic regression. Statistical analyses were performed using SPSS IBM Corporation, Armonk, NY (version 27) with the significance level set at *P* < .05.

## Results

### Study demographics

A total of 352 dentists responded to this survey. After excluding 72 incomplete surveys, 280 responses remained, corresponding to 10.4% of practising dentists in Singapore. The demographic characteristics of the surveyed dentists are summarised in Table 1. The majority (79.6%) of the respondents were under 50 years old, and female (60.4%), consistent with the dentist demographics reported by the Singapore Dental Council.^25^ The majority of respondents (75%) received their basic dental training in Singapore. Compared with the national dental demographics, our sample included a higher proportion of public sector dentists (34.6%) and specialists (32.9%). The increased representation from the public sector likely reflects the 18.2% of participants with 0 to 5 years of experience, many of whom are recent graduates serving as dental officers in the public sector. The higher representation of specialists is likely due to the inclusion of all dentists who had completed basic specialist training, although full specialist accreditation by the Dental Council requires completion of advanced specialist training.

**Table 1:** Characteristics of surveyed dentists practicing in Singapore 2024-2025.

Sex	*n* (%)
Male	111 (39.6)
Female	169 (60.4)
**Age**	
<30 yrs	57 (20.4)
<40 yrs	118 (42.1)
<50 yrs	48 (17.1)
<60 yrs	31 (11.1)
61+ y	21 (7.5)
Declined to answer	5 (1.8)
**Location of basic dental training**	
Singapore	210 (75.0)
Overseas	70 (25.0)
Location of overseas training	
Australia & NZ	40 (57.1)
Hong Kong, Malaysia, Philippines	5 (7.1)
United Kingdom, Ireland & USA	25 (35.7)
**Years of practice**	
0 to 5 yrs	51 (18.2)
6 to 10 yrs	76 (27.1)
11 to 20 yrs	81 (28.9)
21 to 30 yrs	33 (11.8)
30+ yrs	39 (13.9)
**Main practice sector**	
Private practice	183 (65.4)
Public sector	97 (34.6)
**Specialty**	
General dental practitioner	188 (67.1)
Paediatrics dentistry	19 (6.8)
Periodontics	18 (6.4)
Orthodontics	16 (5.7)
Endodontics	15 (5.4)
Prosthodontics	12 (4.3)
Oral maxillofacial surgery/ oral medicine	10 (3.6)
Dental Public Health	2 (1.0)

### Characteristics of antibiotic prescription practices

Table 2 summarises the respondents’ antibiotic prescribing preferences. The prevailing practice was to prescribe antibiotics for 5 days (86.8%), with Amoxicillin and Augmentin being the most commonly prescribed (97.1%). 14.9% reported always or often prescribing multidrug regimens, with the most common combination being amoxicillin and metronidazole (89.0%).

**Table 2:** Characteristics of antibiotic prescribing preferences of surveyed dentists practicing in Singapore.

	*n* (%)
**Q7 Which antibiotic do you prescribe most often?** [*N* = 272]	
Amoxicillin	186 (68.4)
Amoxicillin + clavulanic acid (eg, Augmentin)	86 (31.6)
**Q8 For how many days do you usually prescribe antibiotics?** [*N* = 280]	
1-3 d	17 (6.1)
5 d	243 (86.8)
6-7 d	19(6.8)
10 d	1 (0.4)
**Q9 How often do you prescribe two antibiotics at the same time?** [*N* = 175]	
Always or often	26 (14.9)
Occasionally or rarely	123 (70.3)
Never	26 (14.9)
**Q10 Which two antibiotics do you prescribe at the same time?** [*N* = 118]	
(1) Amoxicillin & (2) Metronidazole	105 (89.0)
(1) Amoxicillin + clavulanic acid (eg, Augmentin) & (2) Metronidazole	12 (10.2)
(1) Amoxicillin & (2) Amoxicillin + clavulanic acid (eg, Augmentin)	1 (0.8)

(%) calculated based on *N* reported for each question.

Responses indicating ‘Do not do procedure’ were excluded.

### Knowledge and prescribing preferences for clinical and nonclinical scenarios

There was substantial variability in dentists’ knowledge and preferences for antibiotic prescribing across clinical scenarios. These scenarios included treatment of infections, prevention of postprocedural infections, and medical indications for preprocedural prophylaxis. Appropriate use ranged from 6.5% to 97.7% (Table 3).

**Table 3:** Knowledge and prescribing preferences of surveyed dentists in Singapore for clinical and nonclinical scenarios.

Survey Question	Please indicate how often you prescribe a course of antibiotics for treating the following clinical indications, in addition to a dental treatment. [*n* (%)]	Always or often	Occasionally or rarely		Never	*N*
Q12-1	Irreversible pulpitis, asymptomatic apical periodontitis. Moderate/Severe symptoms.	11 (4.3)	84 (32.6)		**163 (56.0)**	258
Q12-2	Irreversible pulpitis, symptomatic apical periodontitis. Moderate/Severe symptoms.	53 (20.5)	106 (40.9)		**100 (38.6)**	259
Q12-3	Pulp necrosis with acute apical abscess. Localised swelling without sinus tract in an otherwise healthy patient.	105 (40.1)	109 (41.6)		**48 (18.3)**	262
Q12-4	Pulp necrosis with chronic apical abscess. Localised swelling with a sinus tract.	45 (17.2)	108 (41.4)		**108 (41.4)**	261
Q12-5	Pulp necrosis, with acute apical abscess, and systemic spread present eg, cellulitis.	**255 (97.7)**	4 (1.5)		2 (0.8)	261
Q12-6	Routine prescription of antibiotics after starting RCT for pulp necrosis with symptomatic apical periodontitis.	41 (16.8)	72 (29.5)		**131 (53.7)**	244
Q12-7	Carious pulp exposure when doing a filling.	5 (1.9)	26 (10.0)		**230 (88.1)**	261
Q12-8	Alveolar osteitis (Dry socket).	82 (32.8)	89 (35.6)		**79 (31.6)**	250
Q12-9	Localised periodontal abscess. Symptomatic, no systemic involvement.	66 (24.7)	116 (43.4)		**85 (31.8)**	267
Q12-10	Adjunctive antibiotics with scaling/root debridement for routine periodontitis treatment.	18 (7.7)	126 (54.1)		**89 (38.2)**	233
Q12-11	Pericoronitis. Localised, moderate/severe symptoms, without systemic involvement.	99 (36.3)	110 (40.3)		**64 (23.4)**	273
Q12-12	Reimplantation of avulsed teeth.	**109 (71.2)**	34 (22.2)		10 (6.5)	153
Q12-13	Peri-implant infections.	76 (46.9)	72 (44.4)		**14 (8.6)**	162
	**Please indicate how often you prescribe a course of antibiotics after the following clinical indications to prevent infections**. [*n* (%)]	**Always or often**	**Occasionally or rarely**		**Never**	***N***
Q13-1	Difficult tooth extraction in a healthy person.	39 (15.1)	133 (51.4)		**87 (33.6)**	259
Q13-2	Surgical tooth extraction in a healthy person.	107 (47.1)	85 (37.4)		**35 (15.4)**	227
Q13-3	Surgical removal of impacted third molars, in a healthy person.	146 (71.2)	38 (18.5)		**21 (10.2)**	205
Q13-4	Dental implant placement.	114 (73.5)	31 (20.0)		**10 (6.5)**	155
Q13-5	Extraction of a tooth in a patient on antiresorptive therapy (eg, bisphosphonates, denosumab)	122 (66.7)	48 (26.2)		**13 (7.1)**	183
Q13-6	Extraction of a tooth in a patient with history of head and neck radiotherapy.	**105 (64.8)**	48 (29.6)		9 (5.6)	162
	**For which of the following patients would you prescribe preoperative prophylactic antibiotics before starting invasive dental treatment**	**Yes**		**No**		***N***
Q14-1	Mitral valve prolapse with valvular regurgitation.	148 (52.9)		**132 (47.1)**		280
Q14-2	Unrepaired atrial or ventricular septal defects.	175 (62.5)		**105 (37.5)**		280
Q14-3	Prosthetic joint replacement (eg, hip or total knee replacement).	80 (28.6)		**200 (71.4)**		280
Q14-4	Coronary artery bypass graft (CABG)	58 (20.7)		**222 (79.3)**		280
Q14-5	None of the above	**71 (25.4)**		209 (74.6)		280
	**To further investigate the common pressures experienced by dentists, please indicate if you would prescribe antibiotics in the following clinical scenarios below**. [*n* (%)]	**Always or often**	**Occasionally or rarely**		**Never**	***N***
Q15-1	If your patients request for antibiotics instead of treatment.	55 (19.6)	162 (57.9)		**63 (22.5)**	280
Q15-2	If you are pressed for time and the patient has a localised odontogenic infection, you would prescribe antibiotics and make another appointment for the patient.	75 (26.8)	146 (52.1)		**59 (21.1)**	280
Q15-3	If the local anaesthetic is ineffective and/or the patient has severe symptoms of irreversible pulpitis, you would prescribe antibiotics first before initiating definitive treatment at a later time.	77 (27.5)	123 (43.9)		**80 (28.6)**	280
Q15-4	If you are unable to come to a definitive diagnosis and the patient has symptoms of a likely odontogenic infection (eg, pain at night, pain on percussion), you would prescribe antibiotics.	34 (12.1)	141 (50.4)		**105 (37.5)**	280
Q15-5	How often do you prescribe antibiotics to avoid potential medico-legal conflicts/patient complaints rather than a clinical indication?	19 (6.8)	158 (56.4)		**103 (36.8)**	280

(%) calculated based on *N* reported for each question.

Answers defined as appropriate are in bold.

We included 4 commonly misunderstood medical indications for preprocedural antibiotic prophylaxis (Table 3; Q14). Only 25.4% of respondents correctly identified that none of the 4 listed conditions required antibiotic prophylaxis, and more than half incorrectly identified mitral valve prolapse (52.9%) and unrepaired septal defects (62.5%) as indications, reflecting limited familiarity with the updated AHA guidelines.^17^

When faced with nonclinical pressures such as patient requests, time constraints, difficulties in achieving anaesthesia, or diagnostic uncertainty, 21.2% to 37.5% of dentists self-reported that they would consistently adhere to appropriate practices (Table 3, Q15). Only a small proportion (6.8%) reported prescribing defensively to avoid medico-legal conflicts. Notably, across all nonclinical scenarios, approximately half of the respondents indicated they would prescribe antibiotics at least occasionally to rarely.

### Clinician characteristics associated with appropriate antibiotic prescribing

Considering the variability in appropriate prescribing, we sought to explore the impact of clinician characteristics on prescribing practices (Table 4). Mean appropriate prescribing rates were highest for the restorative scenarios (58.3% ± 28.6%) and for the management of medically complex patients (56.6% ± 18.0%) and lowest for the periodontal scenarios (30.4% ± 37.5%) and oral surgery scenarios (34.0% ± 29.0%), respectively (Table 4). In general, locally trained dentists and those practising in the public sector had higher appropriate prescription rates. Senior dentists with ≥21 years of experience were more likely to prescribe antibiotics inappropriately for the restorative, oral surgery, and periodontal scenarios.

**Table 4:** Appropriate prescription rates for each clinical category.

	Restorative	Oral Surgery	Periodontal	Medically Complex Patients
**Number of responders**	258	270	259	280
**Appropriate prescription rate**	58.3 ± 28.6	34.0 ± 29.0	30.4 ± 37.5	56.6 ± 18.0
**Sex**				
Female	59.9 ± 28.5	33.4 ± 31.1	33.1 ± 39.5	57.5 ± 18.0
Male	56.0 ± 28.7	31.4 ± 31.0	26.3 ± 33.6	55.1 ± 17.7
**Basic dental training**				
Singapore	60.7 ± 28.9*	33.7 ± 32.1	33.7 ± 39.6*	58.7 ± 16.5⁎⁎⁎
Overseas	51.8 ± 26.7	29.1 ± 27.5	21.1 ± 28.3	50.1 ± 20.1
**Place of practice**				
Private sector	49.7 ± 26.5⁎⁎⁎	22.7 ± 25.5⁎⁎⁎	17.8 ± 27.3⁎⁎⁎	52.5 ± 18.5⁎⁎⁎
Public sector	77.0 ± 23.4	53.1 ± 31.5	57.2 ± 41.6	64.2 ± 13.7
**Years of clinical experience**				
< 10 y	64.5 ± 26.5###	34.4 ± 29.3^#^	36.6 ± 38.0^#^	58.4 ± 17.9
11-20 y	62.7 ± 28.3###	41.5 ± 34.5###	32.0 ± 38.7	55.3 ± 16.1
≥ 21 y	39.3 ± 24.0	19.7 ± 25.8	17.9 ± 31.7	52.2 ± 17.7

⁎ *P* < .05.

⁎⁎⁎ *P* < .001. Differences were determined using independent *T*-tests. ^#^*P* < .05,

### *P* < .001, compared to ≥ 21 y of clinical experience. Pairwise differences were determined using One-way Analysis of Variance (ANOVA), with Bonferroni correction for *post hoc* testing.

In multivariable logistic regression, some associations between clinician characteristics and appropriate prescribing in each individual clinical scenario were observed (summarised in Table 5 and detailed in Supplementary Table 2).

**Table 5:** Statistically significant multivariable odds of appropriate prescribing, by clinician characteristic.

Less likely to prescribe appropriately					More likely to prescribe appropriately			
Male	OS university	Clinical experience†	Public sector		Public sector	Clinical experience†	OS university	Male
		0.30		Q12_1	5.43			
		0.14		Q12_2	3.77			
				Q12_3	3.37			
0.47		0.26		Q12_4	6.10			
				Q12_5				
		0.18		Q12_6	4.59			
		0.24		Q12_7				
		0.29		Q12_8	4.76		2.69	
		0.40		Q12_9	5.97			
				Q12_10	3.86			
				Q12_11	5.03			
				Q12_12				
		0.43		Q13_1	4.04		2.57	
				Q13_2	6.24	2.61*		
				Q13_3	10.02			
			0.16	Q13_4				
				Q13_5	6.37		9.79	
	0.32	0.26*	0.34	Q13_6				
	0.49			Q14_1	3.46			
				Q14_2	3.39			
		0.30		Q14_3	2.34			
				Q14_4	5.03	2.67*		
				Q14_5	3.80			
				Q15_1				
				Q15_2				
				Q15_3				
				Q15_4		3.87		
		0.38	0.38	Q15_5				

⁎ Denotes the group with 11-20 years of clinical experience.

† Denotes the group with ≥21 y of clinical experience unless otherwise indicated.

#### Main practice sector

Consistent with the univariate results, dentists from the public sector were more likely to prescribe appropriately for most clinical scenarios to manage and prevent infections (Q12 and Q13), except for dental implant placement (OR = 0.16, *P* < .001) and tooth extraction in a patient with history of head and neck radiotherapy (OR = 0.34, *P* = .015).

#### Clinical experience

Senior dentists with ≥21 years of experience were more likely to prescribe inappropriately, with inappropriate prescribing occurring in roughly one-third of the clinical scenarios. In contrast, those with 11 to 20 years of experience were more likely to avoid unnecessary antibiotics after surgical extractions in healthy patients (OR: 2.61, *P* = .039) and patients with coronary artery bypass graft (OR: 2.67, *P* < .001).

#### Location of basic dental training

Controlling for other clinician characteristics, dentists who received their basic dental training overseas were more likely to practice appropriate prescribing for patients on antiresorptive agents (OR = 9.79, *P* = .030), for postextraction antibiotics in healthy patients (OR = 2.57, *P* = .012), and when managing alveolar osteitis (OR = 2.69, *P* = .007). However, they were more likely to prescribe inappropriately for tooth extractions in patients with a history of head and neck radiotherapy (OR = 0.32, *P* = .014) despite documented efficacy of systemic antibiotics reducing the risk of osteoradionecrosis.^18^

#### Sex

Sex differences were observed for only one scenario; male dentists self-reported they were significantly more likely to inappropriately prescribe when managing pulp necrosis with chronic apical abscesses (OR = 0.47, *P* = .017).

#### Antibiotic prophylaxis prescribing in commonly misunderstood medical indications

For infective endocarditis and prosthetic joint infections prophylaxis (Q14), dentists in the public sector were more likely to prescribe appropriately (OR: 2.34-5.03, *P* < .05). In contrast, senior dentists (≥21 years of experience) were more likely to prescribe prophylactic antibiotics inappropriately for patients with total joint replacements (OR = 0.30, *P* = .001), while dentists trained overseas were more likely to do so for mitral valve prolapse (OR = 0.49, *P* = .025). Dentists with 11 to 20 years of experience were more likely to avoid unnecessary prescriptions for patients with a history of coronary artery bypass grafts (OR = 2.67, *P* = .021).

#### Common nonclinical pressures

When exploring the influence of nonclinical pressures on antibiotic prescribing behaviour (Q15), senior dentists (≥21 years of experience) were less likely to prescribe antibiotics without definitive diagnoses (OR = 3.87, *P* = .004), but were more likely to prescribe defensively to avoid potential medicolegal conflicts and patient complaints (OR = 0.38, *P* = .034). This defensive behaviour was also more likely amongst public sector dentists (OR = 0.38, *P* = .045).

### Knowledge and attitudes towards antimicrobial resistance

Given the critical role dentists play in antibiotic prescriptions and the broader issue of antimicrobial resistance, we sought to understand the knowledge and attitudes towards antimicrobial resistance (Table 6). Although 80% of respondents demonstrated a positive attitude towards antimicrobial resistance, notable gaps in knowledge were observed. More than half (59.3%) were unaware that antimicrobial-resistant bacteria can be easily transmitted, particularly when infection control is compromised.

**Table 6:** Knowledge and attitudes towards antimicrobial resistance of surveyed dentists in Singapore.

	Below are a set of statements regarding antibiotics and antibiotics resistance. Please indicate which of the following statements are correct to the best of your knowledge. [*n* (%)]	True	False	Do not know	*N*
Q16-1	Prescribing antibiotics for longer than the recommended duration does not contribute to antibiotics resistance.	39 (13.9)	**208 (74.3)**	33 (11.8)	280
Q16-2	Antibiotics-resistant bacteria spreads easily from person to person.	**114 (40.7)**	101 (36.1)	65 (23.2)	280
Q16-3	Only immunocompromised people can carry antibiotics-resistant bacteria.	3 (1.0)	**263 (93.9)**	14 (5.0)	280
Q16-4	Poor infection control practices (eg, poor hand hygiene) by healthcare professionals can promote antibiotics resistance.	**114 (40.7)**	107 (38.2)	59 (21.1)	280
	**Below are a set of statements regarding antibiotics resistance. Please indicate to what extent you agree or disagree with each statement.** [*n* (%)]	**Strongly agree or agree**		**Disagree or strongly disagree**	***N***
Q17-1	Inappropriate antibiotics prescriptions in dentistry contribute to antibiotics resistance.	**274 (97.9)**		6 (2.1)	280
Q17-2	Knowledge of the problems associated with antibiotics resistance influences your prescribing practices.	**275 (98.2)**		5 (1.8)	280
Q17-3	Antibiotics resistance is a problem in Singapore.	**233 (83.5)**		46 (16.5)	279

Answers defined as appropriate are in bold.

### Sources of prescribing knowledge and training needs

The sources of prescribing knowledge and training needs of the surveyed dentists are shown in Table 7. Only 25.7% of respondents had previously attended training or courses on this topic. Most (88.2%) indicated interest in further initiatives to improve antibiotic prescribing, with locally developed guidelines (83.6%) and mobile prescribing applications (70.4%) being the most well-received.

**Table 7:** Sources of prescribing knowledge and training needs of surveyed dentists practicing in Singapore.

**Q18 Have you attended any training/educational courses on antibiotics prescribing or stewardship?** [*N* = 280]			***n* (%)**	
		No	208 (74.3)	
		Yes	72 (25.7)	
		If so, when?		
		Last 12 mo	9 (12.5)	
		12 to 36 mo	21 (29.2)	
		3-5 y	14 (19.4)	
		More than 5 y	27 (37.5)	
		Blank	1 (1.4)	
**Q19 What are your two main sources of knowledge on therapeutics and prescriptions?** [*N* = 280]				
Dental-specific therapeutic guidelines			237 (42.3)	
Other drug references (eg, NDF, MIMS)			101 (18.0)	
What has worked for you in the past. (eg, Experience)			95 (17.0)	
Colleagues			86 (15.4)	
Alternative online sources. (eg, Google)			41 (7.3)	
**Q20 Would you like to receive more training in antibiotics use and prescribing?** [*N* = 280]				
Yes			247 (88.2)	
No			33 (11.8)	
**Q21 In your opinion, which of the following measures will be helpful in improving the appropriateness of antibiotics prescribing?**	**Very helpful**	**Moderately or slightly helpful**	**Not helpful**	***N***
Q21-1 Availability of locally developed guidelines for dental prescribing	234 (83.6)	43 (15.4)	3 (1.1)	280
Q21-2 Online e-learning course for CPE	153 (54.6)	121 (43.2)	6 (2.1)	280
Q21-3 Lecture/workshop course for CPE	113 (40.4)	158 (56.4)	9 (3.2)	280
Q21-4 Mobile app for dental prescribing	197 (70.4)	73 (26.1)	10 (3.6)	280
Q21-5 Computer-aided prescribing	149 (53.2)	119 (42.5)	12 (4.3)	280

NDF, National Drug Formulary; MIMS, Monthly Index of Medical Specialties.

## Discussion

This is the first study in Singapore to assess antibiotic prescribing practices, knowledge, and attitudes among dentists. While most dentists recognised the importance of appropriate antibiotic use and the concerns with AMR, inadequate knowledge and suboptimal prescribing practices were reported. This comprehensive survey highlights gaps and misconceptions in the use of antibiotics and in AMR knowledge, and provides a basis for the development of targeted interventions to support appropriate antibiotic prescribing in Singaporean dentistry.

Inappropriate prescribing was most common in the oral surgery, periodontal and implant-related clinical scenarios. In particular, 71.2% of respondents reported always or often prescribing prophylactic antibiotics after the surgical removal of impacted third molars in healthy individuals, despite increasing evidence indicating that such prophylactic antibiotics are unnecessary.^19^, ^20^, ^21^ Additionally, a recent intervention study in Singapore found no increase in postoperative infection rates after reducing antibiotic use for third molar surgery from 84% to 21%.^22^ Likewise, 73.5% of respondents reported routinely (always or often) prescribing prophylactic antibiotics when placing dental implants. While prophylactic antibiotics have been reported to reduce early implant failure,^23^ the absolute risk reduction was only 2.9%. Moreover, for systemically healthy patients, there were no differences in early failure rates.^24^ Thus, prophylactic antibiotics should not be routinely prescribed and should be reserved for surgically or medically complex patients.

Inappropriate antibiotic prescribing for periapical and periodontal abscesses without systemic involvement was common in this study. The observed rates for pulp necrosis with an acute apical abscess (40.1%) and localised periodontal abscesses (24.7%) are comparable to international findings, with reported ranges of 37.9% to 88.7% and 10.0% to 82.7%, respectively, in a recent systematic review.^9^ This persists despite clear, well-established evidence that antibiotics are not recommended in these clinical scenarios.^26^^,^^27^ Together, these findings highlight a persistent global gap between evidence and practice, suggesting that targeted continuing dental education in these specific areas to improve knowledge may be one strategy to improve prescribing practices.

Our multivariable model highlighted groups that may benefit from more targeted interventions. Public-sector dentists demonstrated more appropriate prescribing in the current study. This may reflect the structured continuing education programmes and routine alignment of practices with the dissemination of internal clinical practice guidelines within public healthcare institutions. In contrast, private-sector dentists often have to rely on self-directed learning and independently seek out clinical updates. Similarly, inappropriate prescribing was more prevalent amongst senior dentists with ≥21 years of experience, particularly for antibiotic prophylaxis, an area with multiple practice guidelines revisions over the past decade. Structured channels for updates through professional dental organisations may help improve the dissemination of evolving recommendations.^28^

Differences in prescribing practices were also observed between locally trained and overseas trained dentists, although patterns varied by clinical scenario. These variations likely reflect differences in education curriculum and international prescribing norms. Differences in patient populations and disease prevalence, for example in head and neck cancer, across countries may influence awareness of the appropriate prescription for that scenario. Health systems factors may also play a role, for example in Thailand where antibiotics are available over-the-counter, the broader access may also shape prescribing behaviours and social norms around antibiotic use.^15^ In addition, we also observed several nonclinical factors affecting prescribing practices, including time pressure, patient expectations, concerns about medico-legal dispute, inability to achieve anaesthesia and diagnostic uncertainty, which are consistent with other studies.^29^^,^^30^ For example, a cross-sectional study conducted in Australia reported that time pressure and patient expectations affected the prescribing practices of 77% and 82% of dentists, respectively. Additionally, 67% would prescribe antibiotics routinely or occasionally when a diagnosis could not be made.^4^ Importantly, patients also influence prescribing behaviours and play a critical role in antibiotic stewardship. A 2019 cross-sectional population survey in Singapore showed that individuals with more favourable attitude scores towards antibiotic use were less likely to expect and ask for antibiotics from their primary care doctor.^31^ Increasing public awareness of appropriate antibiotic use may positively influence antibiotic prescribing practices by dentists.

In the current study, the most preferred strategy for improving antibiotic prescribing was the development of local prescribing guidelines for dentists. National guidelines could support more consistent, evidence-based, and rational prescribing practices across Singapore. Notably, the first antibiotic prescribing clinical practice guideline for dentistry was released by the Academy of Medicine Singapore^16^ midway through our data collection period. However, limited awareness was observed among our respondents, underscoring the challenges in guideline dissemination. Moreover, while clinical practice guidelines provide a necessary foundation for appropriate prescribing, evidence from randomized controlled trials indicates that the dissemination of guidelines alone is insufficient for improving antibiotic prescribing practices.^32^, ^33^, ^34^ Behaviour change frameworks such as the Capability, Opportunity, Motivation–Behaviour (COM-B) model to address the complex interplay of factors influencing prescribing practices found in this study, and use of implementation science strategies, could guide the development of future antimicrobial stewardship interventions.^23^ Recent systematic reviews of dental antibiotic stewardship interventions have found audit-based strategies, particularly those incorporating personalised feedback and individualised behaviour change messages to be effective.^33^^,^^35^ However, the existing evidence base remains limited, with considerable heterogeneity in study design and outcome measures, underscoring the need the need for further high-quality research to inform implementation.

Digital resources such as mobile applications with integrated prescribing features were the second most preferred mode of content delivery among the dentists surveyed. Such tools may be especially effective when embedded into clinical workflows, offering timely context-specific support. Qualitative evidence suggests that dentists may experience difficulty interpreting and operationalising guideline recommendations in clinical practice, supporting a role for clinical decision support tools to facilitate their implementation.^34^ For example, a mobile app, Drugs4dent, which offers dental-specific drug information, guideline-aligned prescribing recommendations, drug-interaction and allergy alerts, and patient education resources on antibiotic use, was reported by users to be intuitive and highly clinically relevant.^36^^,^^37^ Together, these observations highlight the importance of aligning the dissemination of updated guidelines and continuing education with dentists’ preferred digital learning modalities over conventional lectures and workshops, and embedding them within multimodal antimicrobial stewardship strategies to improve antibiotic prescribing.

Our study uniquely examines dentists’ knowledge of AMR, which has not been assessed in previous surveys. In general, the surveyed dentists demonstrated a positive attitude towards antibiotic stewardship and were receptive to additional training. However, knowledge on antimicrobial resistance mechanisms was consistently poor across all groups, regardless of years of experience or practice sector. This discrepancy may reflect the growing prominence of AMR in global health, highlighted by the intensified efforts by the World Health Organisation over the past 5 years.^38^^,^^39^ It may also stem from longstanding deficiencies in AMR-related teaching within dental education, where a dentistry-specific AMR curriculum has yet to be formally developed or evaluated.^40^ Incorporating the latest understanding of how AMR develops and spreads, and the rationale behind optimal doses and duration, both at the undergraduate level and ongoing professional development may also contribute to better informed prescribing decisions and support effective antibiotic stewardship.

This study has some limitations. We used a convenience sampling method, distributing the online survey via email mailing lists where the total number of dentists who received the survey is unknown, and the response rate cannot be calculated. Nonresponse bias is possible as dentists with a stronger interest in antibiotic stewardship may have been more likely to participate in the survey. While such a bias may lead to an over-representation of appropriate prescribing practices, we note that the demographic characteristics of our respondents were broadly consistent with the national dentist demographics reported by the Singapore Dental Council,^25^ suggesting some degree of comparability with the wider dentist population in Singapore. Nevertheless, the findings should be interpreted with caution as they may reflect the views and practices of dentists who are more engaged with antibiotic stewardship matters. Our study received fewer responses than the targeted sample size of 337. However, at *n* = 280 and prevalence = 50%, the 95% margin of error is approximately 5.5% with finite population correction. While precision is modestly lower than planned, the estimates remain reasonably precise for population-level inference.

Another limitation is the inability to compare differences in prescribing practices, knowledge and attitudes between general dentists and specialists, and between the individual specialties. Specialists form a small proportion of the dental workforce in Singapore, with only 50 to 80 registered specialists in most specialties.^25^ When analysed collectively as a single group comprising all specialties, higher rates of inappropriate prescribing were observed. However, this finding should be interpreted with caution as specialists, being subject matter experts within their own fields, may be less familiar with the updated prescribing practices outside their specialty. In addition, specialists may manage patients with more complex medical histories and multiple comorbidities, for whom antibiotic use may be clinically indicated for reasons not captured by the questionnaire. Similarly, the lower rates of appropriate prescribing observed among dentists with more than 21 years of experience may reflect differences in case complexity rather than knowledge deficits. As such, the aggregated findings may not accurately represent the diversity of prescribing behaviours across different specialties and case complexities.

Nevertheless, our study provides the first insight into the antibiotic prescribing practices of dentists in Singapore, highlighting key clinical scenarios that need to be addressed in education and training, and highlighting the urgent need for continuing education in antibiotic prescribing. This study highlights key areas where targeted interventions can enhance antibiotic stewardship in dentistry. These efforts seek to address knowledge gaps in specific clinical areas, align with dentists’ preferred mode of content delivery, and engage patients whose attitudes and expectations significantly influence prescribing decisions.

## Conclusions

Advancing the overarching goal of implementation of antibiotic stewardship strategies requires a clear understanding of the factors contributing to inappropriate prescribing. The knowledge provided by this study will help guide future targeted interventions to promote appropriate prescribing practices across the dental profession in Singapore.

Despite widespread recognition of AMR as a major threat, many dentists still demonstrated gaps in their understanding of AMR and inappropriate prescribing. Improved dissemination of updated local guidelines and expanded access to digital learning opportunities may support consistent, rational antibiotic prescribing practices. Strengthening foundational knowledge at the undergraduate level and through continuing education is also important. Nearly all respondents expressed a strong interest in further training, underscoring both the need and timeliness of developing a targeted educational intervention to improve antibiotic prescribing.

## CRediT authorship contribution statement

**Jia Qi Low:** Writing – original draft, Writing – review & editing. **Marietta Taylor:** Data curation, Formal analysis, Writing – review & editing. **Behnjemyn Jeng Kit Loh:** Investigation, Methodology, Writing – review & editing. **Jacob Ren Jie Chew:** Data curation, Formal analysis, Funding acquisition, Investigation, Methodology, Writing – review & editing. **Leanne Teoh:** Conceptualization, Methodology, Supervision, Writing – review & editing. **Charlene Enhui Goh:** Conceptualization, Funding acquisition, Investigation, Methodology, Supervision, Writing – review & editing.

## Conflict of interest

L.T. is recognised as an inventor of MIMS Drugs4dent at the University of Melbourne. MIMS Drugs4dent is a commercial product, and L.T. will receive royalties in the future. All other authors report no conflicts of interest.

## Appendix Supplementary materials

Image, application 1

## Footnotes

Supplementary material associated with this article can be found, in the online version, at doi:10.1016/j.identj.2026.109654.
