# Supplementary material for: Dental Antibiotic Prescribing Practices and Antimicrobial Resistance Knowledge in Singapore
Source: Int Dent J. 2026 Jun 3;76(4):109654. doi: 10.1016/j.identj.2026.109654 (PMC13254855; doi:10.1016/j.identj.2026.109654)
Supplement: Supplementary file 1 [file mmc1.docx]

**Supplementary Table 1.** Survey questions used

| **Question** | **Content** | |
| --- | --- | --- |
| Q1 | What is your age? | |
| Q2 | What is your sex? | |
|  | Male | |
|  | Female | |
| Q3 | Where did you complete your basic dental training? If "Overseas", please state the country. | |
|  | Singapore | |
|  | Overseas | |
| Q4 | Where do you predominantly practice dentistry? | |
|  | Private practice | |
|  | Public sector | |
| Q5 | How many years have you been practicing dentistry? | |
|  | 0-5 years | |
|  | 6-10 years | |
|  | 11-20 years | |
|  | 21-30 years | |
|  | More than 30 years | |
| Q6 | What is your specialty? | |
|  | General Dental Practitioner | |
|  | Endodontics | |
|  | Oral & Maxillofacial Surgery / Oral Medicine | |
|  | Orthodontics | |
|  | Paediatrics Dentistry | |
|  | Periodontics | |
|  | Prosthodontics | |
|  | Dental Public Health | |
| Q7 | Select the antibiotics that you prescribe MOST often? Select ONE antibiotic only. | |
|  | Penicillin V | |
|  | Amoxicillin | |
|  | Amoxicillin + clavulanic acid (e.g. Augmentin) | |
|  | Metronidazole | |
|  | Clindamycin | |
|  | Erythromycin | |
|  | Azithromycin | |
|  | Clarithromycin | |
|  | Ciprofloxacin | |
|  | Moxifloxacin | |
|  | Cefalexin | |
|  | Doxycycline | |
|  | Others | |
| Q8 | For how many days do you usually prescribe antibiotics? | |
| Q9 | How often do you prescribe two antibiotics at the same time? (E.g., amoxicillin + metronidazole) | |
|  | Always | |
|  | Often | |
|  | Occasionally | |
|  | Rarely | |
|  | Never | |
| Q10 | Which two antibiotics do you prescribe at the same time? | |
|  | Penicillin V | |
|  | Amoxicillin | |
|  | Amoxicillin + clavulanic acid (e.g. Augmentin) | |
|  | Metronidazole | |
|  | Clindamycin | |
|  | Erythromycin | |
|  | Azithromycin | |
|  | Clarithromycin | |
|  | Ciprofloxacin | |
|  | Moxifloxacin | |
|  | Cefalexin | |
|  | Doxycycline | |
|  | Others | |
| Q11 | For what clinical indications do you usually prescribe combination antibiotics? | |
| Q12 | Please indicate how often you prescribe a course of antibiotics for treating the following clinical indications, in addition to a dental treatment. | |
|  | Always | |
|  | Often | |
|  | Occasionally | |
|  | Rarely | |
|  | Never | |
|  | Do not do procedure | |
| Q12_1 (R) | Irreversible pulpitis, asymptomatic apical periodontitis. Moderate/Severe symptoms | |
| Q12_2 (R) | Irreversible pulpitis, symptomatic apical periodontitis. Moderate/Severe symptoms | |
| Q12_3 (R) | Pulp necrosis with acute apical abscess. Localised swelling without sinus tract in an otherwise healthy patient | |
| Q12_4 (R) | Pulp necrosis with chronic apical abscess. Localised swelling with a sinus tract | |
| Q12_5 (R) | Pulp necrosis, with acute apical abscess, and systemic spread present. E.g., cellulitis | |
| Q12_6 (R) | Routine prescription of antibiotics after starting RCT for pulp necrosis with symptomatic apical periodontitis | |
| Q12_7 (R) | Carious pulp exposure when doing a filling | |
| Q12_8 (O) | Alveolar osteitis (Dry socket) | |
| Q12_9 (P) | Localised periodontal abscess. Symptomatic, no systemic involvement | |
| Q12_10 (P) | Adjunctive antibiotics with scaling/root debridement for routine periodontitis treatment | |
| Q12_11 (O) | Pericoronitis. Localised, moderate/severe symptoms, without systemic involvement | |
| Q12_12 (O) | Reimplantation of avulsed teeth | |
| Q12_13 (P) | Peri-implant infections | |
| Q13 | Please indicate how often you prescribe a course of antibiotics after the following clinical indications to prevent infections. | |
|  | Always | |
|  | Often | |
|  | Occasionally | |
|  | Rarely | |
|  | Never | |
|  | Do not do procedure | |
| Q13_1 (O) | Difficult tooth extraction in a healthy person | |
| Q13_2 (O) | Surgical tooth extraction in a healthy person | |
| Q13_3 (O) | Surgical removal of impacted 3rd molars, in a healthy person | |
| Q13_4 (P) | Dental implant placement | |
| Q13_5 (M) | Extraction of a tooth in a patient on anti-resorptive therapy. E.g., bisphosphonates, denosumab | |
| Q13_6 (M) | Extraction of a tooth in a patient with history of head and neck radiotherapy | |
| Q14 | For which of the following patients would you prescribe pre-operative prophylactic antibiotics before starting invasive dental treatment (e.g. manipulation of the gingival tissues, periapical region of teeth, or perforation of the oral mucosa) to prevent infective endocarditis or joint infections? (Please select all that apply) | |
|  | Yes | |
|  | No | |
| Q14_1 (M) | Mitral valve prolapse with valvular regurgitation | |
| Q14_2 (M) | Unrepaired atrial or ventricular septal defects | |
| Q14_3 (M) | Prosthetic joint replacement. E.g., hip or total knee replacement | |
| Q14_4 (M) | Coronary artery bypass graft (CABG) | |
| Q14_5 (M) | None of the above | |
| Q15 | To further investigate the common pressures experienced by dentists, please indicate if you would prescribe antibiotics in the following clinical scenarios below. | |
|  | Always | |
|  | Often | |
|  | Occasionally | |
|  | Rarely | |
|  | Never | |
| Q15_1 | If your patients request for antibiotics instead of treatment. | |
| Q15_2 | If you are pressed for time and the patient has a localised odontogenic infection, you would prescribe antibiotics and make another appointment for the patient. | |
| Q15_3 | If the local anaesthetic is ineffective and/or the patient has severe symptoms of irreversible pulpitis, you would prescribe antibiotics first before initiating definitive treatment at a later time. | |
| Q15_4 | If you are unable to come to a definitive diagnosis and the patient has symptoms of a likely odontogenic infection (e.g., pain at night, pain on percussion), you would prescribe antibiotics. | |
| Q15_5 | How often do you prescribe antibiotics to avoid potential medico-legal conflicts/patient complaints rather than a clinical indication? | |
| Q16 | Below are a set of statements regarding antibiotics and antibiotics resistance. Please indicate which of the following statements are correct to the best of your knowledge. | |
|  | True | |
|  | False | |
|  | Do not know | |
| Q16_1 | Prescribing antibiotics for longer than the recommended duration does not contribute to antibiotics resistance. | |
| Q16_2 | Antibiotics-resistant bacteria spreads easily from person to person. | |
| Q16_3 | Only immunocompromised people can carry antibiotics-resistant bacteria. | |
| Q16_4 | Poor infection control practices (e.g., poor hand hygiene) by healthcare professionals can promote antibiotics resistance. | |
| Q17 | Below are a set of statements regarding antibiotics resistance. Please indicate to what extent you agree or disagree with each statement. | |
|  | Strongly Agree | |
|  | Agree | |
|  | Disagree | |
|  | Strongly Disagree | |
| Q17_1 | Inappropriate antibiotics prescriptions in dentistry contribute to antibiotics resistance. | |
| Q17_2 | Knowledge of the problems associated with antibiotics resistance influences your prescribing practices. | |
| Q17_3 | Antibiotics resistance is a problem in Singapore. | |
| Q18 | Have you attended any training/educational courses on antibiotics prescribing or stewardship? | |
|  | Yes. If yes, when? | |
|  | Last 12 months | |
|  | 12-36 months | |
|  | 3-5 years | |
|  | More than 5 years | |
|  | No | |
| Q19A | What are your two main sources of knowledge on therapeutics and prescriptions? | |
|  |  | Colleagues |
|  |  | What has worked for you in the past, i.e., experience |
|  |  | Dental-specific therapeutic guidelines |
|  |  | Other drug references - National Drug Formulatory (NDF), MIMS |
|  |  | Alternative online sources, e.g., Google |
| Q19B | Please rank the two most common sources, with '1' being the most common and '2' being the least common. | |
| Q20 | Would you like to receive more training in antibiotics use and prescribing? | |
|  | Yes | |
|  | No | |
| Q21 | In your opinion, which of the following measures will be helpful in improving the appropriateness of antibiotics prescribing? | |
|  | Very Helpful | |
|  | Moderately Helpful | |
|  | Slightly Helpful | |
|  | Not Helpful | |
| Q21_1 |  | Availability of locally developed guidelines for dental prescribing |
| Q21_2 |  | Online e-learning course for CPE |
| Q21_3 |  | Lecture/workshop course for CPE |
| Q21_4 |  | Mobile app for dental prescribing |
| Q21_5 |  | Computer-aided prescribing |

The clinical context for the clinical scenarios (Q12 to Q14) are restorative (R), oral surgery (O), periodontal and implant-related (P), and the management of medically complex patients (M)

## **Supplementary Table 2.** Statistically significant multivariable logistic regression the odds of appropriate prescribing in surveyed dentists in Singapore.

Accounting for sex, location of dental degree, practice sector, and years of clinical experience (<10, 11 to 20, 21+ years)

|  | **OR** | ***p*-value** | **95%CI Lower** | **95%CI Upper** |
| --- | --- | --- | --- | --- |
| **Q12-1: Irreversible pulpitis, asymptomatic apical periodontitis; *Moderate/Severe symptoms*** | | | | |
| Public sector (ref Private sector) | 5.43 | 0.000 | 2.43 | 12.14 |
| Clinical experience (ref <10 years) |  |  |  |  |
| 21+ years | 0.30 | 0.001 | 0.15 | 0.59 |
| **Q12-2: Irreversible pulpitis, symptomatic apical periodontitis, Moderate/Severe symptoms** | | | | |
| Public sector (ref Private sector) | 3.77 | 0.000 | 1.89 | 7.54 |
| Clinical experience (ref <10 years) |  |  |  |  |
| 21+ years | 0.14 | 0.000 | 0.06 | 0.35 |
| **Q12-3: Pulp necrosis with acute apical abscess. Localised swelling without sinus tract** | | | | |
| Public sector (ref Private sector) | 3.37 | 0.002 | 1.56 | 7.29 |
| **Q12_4: Pulp necrosis with chronic apical abscess; localised swelling with a sinus tract** | | | | |
| Male (ref Female) | 0.47 | 0.017 | 0.25 | 0.87 |
| Public sector (ref Private sector) | 6.10 | 0.000 | 3.01 | 12.36 |
| Clinical experience (ref <10 years) |  |  |  |  |
| 21+ years | 0.26 | 0.001 | 0.12 | 0.57 |
| **Q12_6: Routine prescription of antibiotics after starting RCT for pulp necrosis with symptomatic apical periodontitis.** | | | | |
| Public sector (ref Private sector) | 4.59 | 0.000 | 2.17 | 9.71 |
| Clinical experience (ref <10 years) |  |  |  |  |
| 21+ years | 0.18 | 0.000 | 0.08 | 0.38 |
| **Q12_7: Carious pulp exposure when doing a filling.** | | | | |
| Clinical experience (ref <10 years) |  |  |  |  |
| 21+ years | 0.24 | 0.004 | 0.09 | 0.63 |
| **Q12_8: Alveolar osteitis (Dry socket)** | | | | |
| OS degree (ref Singapore degree) | 2.69 | 0.007 | 1.31 | 5.56 |
| Public sector (ref Private sector) | 4.76 | 0.000 | 2.37 | 9.57 |
| Clinical experience (ref <10 years) |  |  |  |  |
| 21+ years | 0.29 | 0.009 | 0.11 | 0.73 |
| **Q12_9: Localised periodontal abscess, symptomatic, no systemic involvement.** | | | | |
| Public sector (ref Private sector) | 5.97 | 0.000 | 3.09 | 11.52 |
| Clinical experience (ref <10 years) |  |  |  |  |
| 21+ years | 0.40 | 0.039 | 0.17 | 0.95 |
| **Q12_10: Adjunctive antibiotics with scaling/root debridement for periodontitis treatment.** | | | | |
| Public sector (ref Private sector) | 3.86 | 0.000 | 1.87 | 7.95 |
| **Q12_11: Pericoronitis Localised, moderate/severe symptoms, without systemic involvement.** | | | | |
| Public sector (ref Private sector) | 5.03 | 0.000 | 2.53 | 10.01 |
| **Q13_1: Difficult tooth extraction in a healthy person.** | | | | |
| OS degree (ref Singapore degree) | 2.57 | 0.012 | 1.23 | 5.38 |
| Public sector (ref Private sector) | 4.04 | 0.000 | 2.03 | 8.04 |
| Clinical experience (ref <10 years) |  |  |  |  |
| 21+ years | 0.43 | 0.048 | 0.19 | 0.99 |
| **Q13_2: Surgical tooth extraction in a healthy person.** | | | | |
| Public sector (ref Private sector) | 6.24 | 0.000 | 2.51 | 15.51 |
| Clinical experience (ref <10 years) |  |  |  |  |
| 11 to 20 years | 2.61 | 0.039 | 1.05 | 6.46 |
| **Q13_3: Surgical removal of impacted 3rd molars, in a healthy person** | | | | |
| Public sector (ref Private sector) | 10.02 | 0.000 | 3.11 | 32.29 |
| **Q13_4:** Dental implant placement | | | | |
| Public sector (ref Private sector) | 0.16 | 0.000 | 0.06 | 0.39 |
| **Q13_5: Extraction of a tooth in a patient on anti-resorptive therapy** | | | | |
| Public sector (ref Private sector) | 6.37 | 0.042 | 1.07 | 37.8 |
| OS degree (ref Singapore degree) | 9.79 | 0.030 | 1.67 | 57.4 |
| **Q13_6: Extraction of a tooth in a patient with history of head and neck radiotherapy** | | | | |
| Public sector (ref Private sector) | 0.34 | 0.015 | 0.14 | 0.81 |
| OS degree (ref Singapore degree) | 0.32 | 0.014 | 0.13 | 0.80 |
| Clinical experience (ref <10 years) |  |  |  |  |
| 11 to 20 years | 0.26 | 0.002 | 0.11 | 0.62 |
| **Q14_1: Mitral valve prolapse with valvular regurgitation** | | | | |
| OS degree (ref Singapore degree) | 0.49 | 0.025 | 0.26 | 0.91 |
| Public sector (ref Private sector) | 3.46 | 0.000 | 1.93 | 6.21 |
| **Q14_2: Unrepaired atrial or ventricular septal defects** | | | | |
| Public sector (ref Private sector) | 3.39 | 0.000 | 1.89 | 6.09 |
| **Q14_3: Prosthetic joint replacement E.g., hip or total knee replacement** | | | | |
| Public sector (ref Private sector) | 2.34 | 0.014 | 1.19 | 4.61 |
| Clinical experience (ref <10 years) |  |  |  |  |
| 21+ years | 0.30 | 0.001 | 0.15 | 0.60 |
| **Q14_4: Coronary artery bypass graft (CABG)** | | | | |
| Public sector (ref Private sector) | 5.03 | 0.000 | 1.96 | 12.9 |
| Clinical experience (ref <10 years) |  |  |  |  |
| 11 to 20 years | 2.67 | 0.021 | 1.16 | 6.16 |
| **Q14_5: None of the above** | | | | |
| Public sector (ref Private sector) | 3.80 | 0.000 | 2.00 | 7.19 |
| **Q15_4: No definitive diagnosis, symptoms of a likely odontogenic infection**. | | | | |
| Clinical experience (ref <10 years) |  |  |  |  |
| 21+ years | 3.87 | 0.004 | 1.56 | 9.60 |
| **Q15_5: To avoid potential medico-legal conflicts/patient complaints**. | | | | |
| Public sector (ref Private sector) | 0.38 | 0.045 | 0.15 | 0.98 |
| Clinical experience (ref <10 years) |  |  |  |  |
| 21+ years | 0.38 | 0.034 | 0.16 | 0.93 |

Appropriate prescribing was defined as answering ‘never’ to the above questions, with the exception of Q12-5, Q12-12, Q13-6 where the appropriate answer was ‘Always/often’

**Supplementary Table 3.** Appropriate prescribing for clinical and non-clinical scenarios and their references

| **Question** | **Appropriate prescribing response** | **Reference** |
| --- | --- | --- |
| Q12-1 to Q12-4, Q12-6, Q12-8 | Never | Teoh et al 2019 |
| Q12-5, Q12-12 | Always or often | Teoh et al 2019 |
| Q12-7, Q12-9, Q12-10, Q12-13, Q13-4, Q13-5 | Never | Local guidelines ^1^ |
| Q12-11 | Never | Schmidt et al 2021 |
| Q13-1 to Q13-3 | Never | Yong et al 2025 |
| Q13-6 | Always or often | Quah et al 2024 |
| Q14-1 to Q14-4 | No | Wilson et al 2021 |
| Q14-5 | Yes | Wilson et al 2021 |
| Q15-1 to Q15-5 | Never | Vazquez-Cancela et al 2024 |

^1^ Singapore CoDS, Academy of Medicine S. Use of Antibiotics in Dentistry. Singapore; 2024
